# Body mass index and albumin levels are prognostic factors for long-term survival in elders with limited performance status

**DOI:** 10.18632/aging.102642

**Published:** 2020-01-16

**Authors:** Kuan-Yu Lai, Tai-Hsien Wu, Chiu-Shong Liu, Chih-Hsueh Lin, Cheng-Chieh Lin, Ming-May Lai, Wen-Yuan Lin

**Affiliations:** 1 Department of Family Medicine, China Medical University Hospital, Taichung City, Taiwan; 2 Department of Family Medicine, College of Medicine, China Medical University, Taichung City, Taiwan; 3 Department of Medical Research, China Medical University Hospital, Taichung City, Taiwan; 4 Department of Social Medicine, College of Medicine, China Medical University, Taichung City, Taiwan

**Keywords:** albumin, body mass index, elder, mortality, performance status

## Abstract

Elderly long-term care facility residents typically have musculoskeletal conditions that may lead to long-term disability and increased mortality. Our main objective was to explore the relationship between body mass index (BMI), albumin levels, and mortality in elderly individuals with limited performance status. Among 182 participants (mean age, 78.8 years; 57% women), 11%, 64%, and 25% had serum albumin levels of <2.8, 2.8-3.5, and >3.5 g/dL, respectively. After multivariate adjustments, diastolic blood pressure >90 mmHg was associated with all-cause mortality [hazard ratio (HR) = 2.08, 95% confidence interval (CI) = 1.13-3.82; P = 0.018]. In addition, BMI <18.5 kg/m2 and albumin level <2.8 g/dL associated with higher mortality than BMI = 18.5-24 kg/m2 and albumin level > 3.5 g/dL (HR = 1.80, 95% CI = 1.11-2.94 and HR = 2.54, 95% CI 1.22-5.30, respectively; P = 0.018 and 0.013, respectively). Highest mortality was noted in participants with albumin levels <2.8 g/dL and BMIs <18.5 kg/m2 (HR = 6.12, 95% CI = 1.85-20.21, P = 0.003). Combined hypoalbuminemia (albumin level < 2.8 g/dL) and low BMI (<18.5 kg/m2) may be a useful prognostic indicator of high mortality risk in elderly individuals with limited performance status.

## INTRODUCTION

Body mass index (BMI) is a simple anthropometric measure of nutritional status, but it is also an important mortality indicator among hospitalized patients [1]. In the elderly population, low BMI, which was defined as a BMI <18.5 kg/m^2^, is more related to increased mortality than is being overweight [2, 3].

The serum albumin level is another important variable for assessing the nutritional status of patients with acute or chronic illness [4]. Hypoalbuminemia, defined as serum albumin levels <3.5 g/dL, is common among hospitalized patients [5] and is associated with mortality and increased risk for various diseases [6], including heart failure [7], cirrhosis [8], and nephrotic syndrome [9]. In addition, poor functional recovery from stroke was noted in a population of patients with low BMIs and serum albumin levels [10]. This suggests, the combination of BMI and serum or urine albumin levels may be predictive of all-cause mortality in the general population [11].

Aging is a major risk factor adversely affecting life expectancy among the elderly [12, 13]. Many of these individuals have a limited performance status (Eastern Cooperative Oncology Group (ECOG) [14] score >2) and use a wheelchair or are bedridden in long-term facilities. Indexes such as the Minimum Data Set (including: frailty index, nutrition, physical function and cognitive function) have been used to predict mortality in nursing homes [15–17]. However, most of those studies have focused on short-term mortality and used qualitative prognostic information instead of quantitative data [18]. We evaluated the utility of the combination of serum albumin and BMI for predicting mortality in elderly residents with limited performance status in long-term care facilities (ECOG score >2) and assessed whether a cut-off value could assist physicians in clinical settings.

## RESULTS

**Figure 1:**
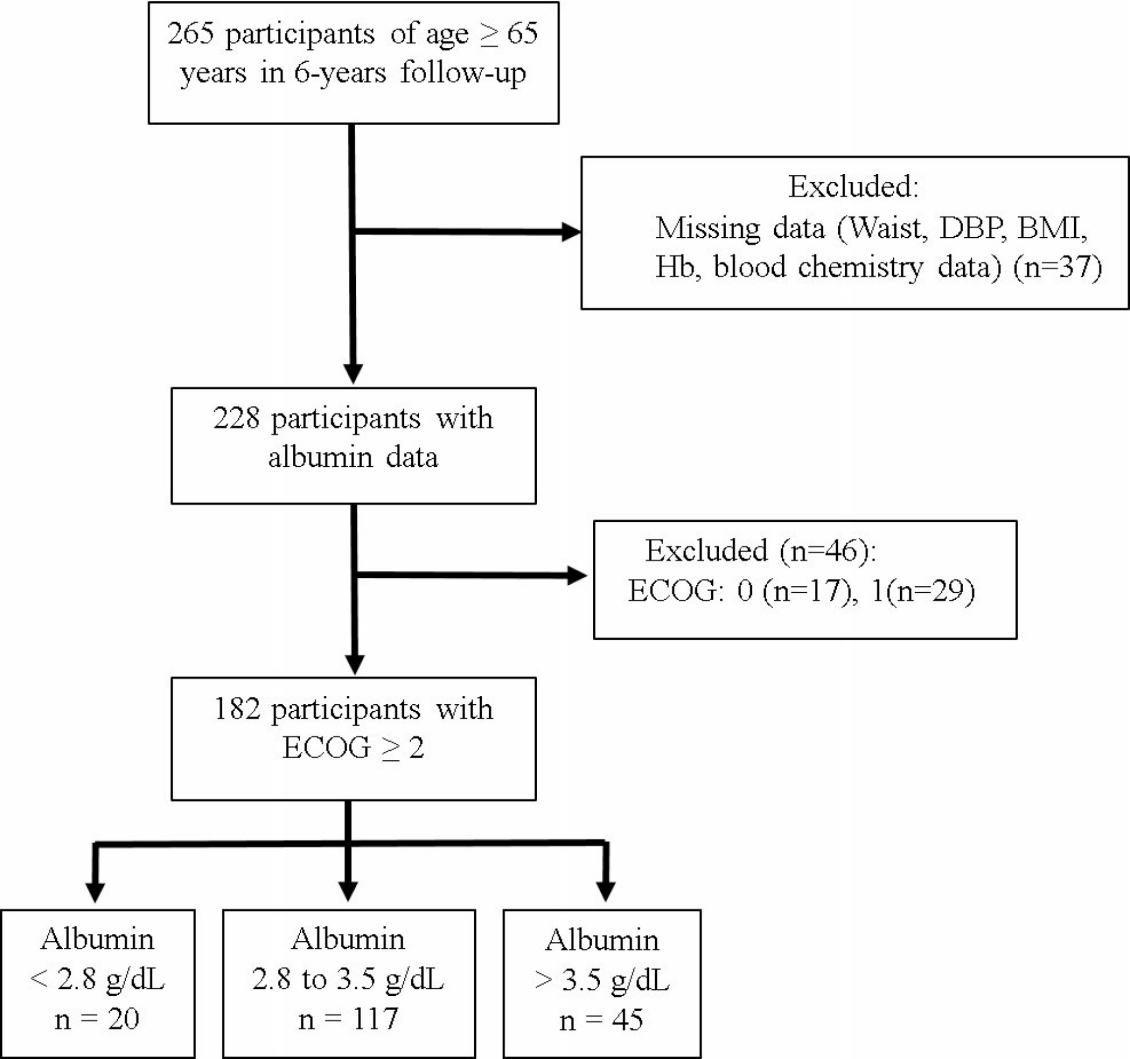
**Study sample.**

In total, 228 individuals (42% men) living in eight different long-term care facilities met our inclusion criteria for BMI and serum albumin at the end of a 6-year follow-up period. Among those, 46 (20%) were excluded due to ECOG scores <2 (Figure 1). Of the remaining 182 participants, 20 (11%) had serum albumin levels <2.8 g/dL, while 117 (64%) had serum albumin levels of 2.8–3.5 g/dL and 45 (25%) had levels >3.5 g/dL. Table 1 lists the basic characteristics of the participants segregated based on their albumin levels. The total number of deaths during follow-up was 139 (76%). The mean age of the participants with an ECOG score ≥2 was 78.8 years (57% women). Compared with the other two groups, participants with albumin levels <2.8 g/dL were older and had a significantly lower waist circumference (WC) and BMI as well as lower hemoglobin, albumin, total cholesterol, and triglyceride (TG) levels.

**Table 1:** Comparison of population characteristics in different serum albumin concentrations

**Variable**	**Study group (n=182)**	**Albumin (g/dL)**			*P
		**<2.8 (n=20)**	**2.8-3.5 (n=117)**	**>3.5 (n=45)**	
Age, years	78.8 ± 7.5	83.6 ± 6.5	78.6 ± 7.5	77.1 ± 7.2	**0.0048**
Female sex; % (n)	57 (103)	7 (7)	64 (66)	29 (30)	0.06
Death; % (n)	76 (139)	14 (19)	64 (89)	22 (31)	0.07
Systolic BP, mmHg	127.0 ± 13.3	130.2 ± 12.2	126.4 ± 12.9	127.3 ± 14.7	0.5
Diastolic BP, mmHg	75.3 ± 8.6	77.6 ± 7.5	75.2 ± 8.3	74.8 ± 9.9	0.4
Waist circumference, cm	80.9 ± 10.1	74.2 ± 9.9	80.5 ± 9.6	84.7 ± 10.0	**0.0004**
Body mass index, kg/m^2^	21.3 ± 4.1	18.3 ± 2.8	21.0 ± 3.	23.1 ± 4.3	**<0.0001**
Biochemistry					
Hemoglobin, g/dL	12.1 ± 1.9	10.6 ± 1.2	11.9 ± 1.8	13.1 ± 1.7	**<0.0001**
Albumin, g/dL	3.2 ± 0.4	2.5 ± 0.3	3.2 ± 0.2	3.7 ± 0.2	**<0.0001**
Glucose, mg/dL	101.0 ± 29.3	95.8 ± 16.0	98.7 ± 21.1	109.2 ± 46.3	0.08
Total cholesterol, mg/dL	178.4 ± 42.6	149.2 ± 36.1	175.5 ± 38.7	198.9 ± 46.1	**<0.0001**
Triglyceride, mg/dL	105.4 ± 108.1	79.1 ± 33.8	95.1 ± 63.4	143.9 ± 186.8	**0.018**
High density lipoprotein, mg/dL	40.2 ± 11.0	36.6 ± 11.1	40.5 ± 10.7	41.1 ± 11.6	0.3
Urin acid, mg/dL	5.3 ± 1.7	4.8 ± 1.5	5.1 ± 1.5	5.7 ± 2.1	0.06
Blood urine nitrogen, mg/dL	16.6 ± 8.3	17.8 ± 8.5	16.3 ± 8.9	16.6 ± 6.4	0.8
Creatinine, mg/dL	1.0 ± 0.5	1.0 ± 0.4	1.1 ± 0.6	1.0 ± 0.3	0.9
Musculoskeletal conditions					
ECOG; % (n)					0.2
2	25 (45)	9 (4)	62 (28)	29 (13)	
3	45 (81)	9 (7)	62 (50)	30 (24)	
4	31 (56)	16 (9)	70 (39)	14 (8)	

BP: Blood Pressure; ECOG: Eastern Cooperative Oncology Group,

*ANOVA for continuous variables, chi-square test for categorical variables for the different albumin concentration

In a univariate analysis, age, diastolic blood pressure (DBP), nutrition indices (i.e., WC, BMI, and albumin level), and renal function indices (i.e., blood urea nitrogen [BUN] and creatinine levels) were all associated with all-cause mortality (*P* <0.05 for all; Table 2). In a multivariate analysis, participants with DBPs ≥90 mmHg had shorter survival times than those with DBPs <90 mmHg (hazard ratio [HR] = 2.08; 95% confidence interval [CI] = 1.13-3.82; *P* = 0.018; Table 2). Moreover, mortality was higher among participants with BMIs <18.5 kg/m^2^ and albumin levels <2.8 g/dL than among those with BMIs of 18.5-24 kg/m^2^ and albumin levels >3.5 g/dL (HR = 1.80, 95% CI = 1.11-2.94 and HR = 2.54, 95% CI 1.22-5.30, respectively; *P* = 0.018 and 0.013, respectively).

**Table 2:** Cox regression models of mortality in elderly participants based on each covariate

**Individual covariates**	**Univariate analysis**				**Multivariate analysis**		
	**HR**	**95% CI**	***P***		**HR**	**95% CI**	***P***
Age, year	**1.04**	**1.02-1.07**	**0.0002**		1.03	1.00-1.06	0.089
Gender Men vs women	1.16	0.83-1.63	0.377		1.10	0.66-1.85	0.709
Systolic BP, mmHg							
<120	Reference				Reference	
120-140	1.39	0.95-2.03	0.087		1.43	0.86-2.38	0.17
≥140	1.20	0.75-1.92	0.449		1.17	0.63-2.17	0.62
Diastolic BP, mmHg							
<80	Reference				Reference		
80-90	**0.59**	**0.36-0.97**	**0.037**		0.82	0.42-1.59	0.547
≥90	0.56	0.28-1.15	0.115		**2.08**	**1.13-3.82**	**0.018**
Waist circumference, cm ≥90/80 vs <90/80*	**0.70**	**0.49-0.99**	**0.042**		0.84	0.47-1.48	0.539
BMI, kg/m^2^							
18.5-24	Reference				Reference		
<18.5	**1.81**	**1.22-2.68**	**0.003**		**1.80**	**1.11-2.94**	**0.018**
24-27	1.03	0.66-1.61	0.909		1.23	0.68-2.21	0.497
≥27	0.44	0.19-1.01	0.052		0.39	0.14-1.07	0.068
Hemoglobin. g/dL <13.7/11.1 vs ≥13.7/11.1*	1.39	1.00-1.95	0.051		1.28	0.79-2.06	0.317
Albumin, g/dL							
>3.5	Reference				Reference		
2.8-3.5	1.35	0.89-2.03	0.155		1.11	0.67-1.84	0.686
<2.8	**3.44**	**1.93-6.14**	**<0.0001**		**2.54**	**1.22-5.30**	**0.013**
Fasting glucose, mg/dL							
<100	Reference				Reference		
100-126	1.04	0.70-1.53	0.853		1.25	0.80-1.94	0.331
≥126	0.89	0.52-1.54	0.679		1.11	0.56-2.21	0.759
Total cholesterol, mg/dL >200 vs ≤200	0.84	0.57-1.24	0.378		1.02	0.64-1.63	0.94
Triglyceride, mg/dL ≥150 vs <150	1.08	0.69-1.67	0.741		1.76	0.98-3.17	0.061
HDL-C, mg/dL ≤40/50 vs >40/50*	0.98	0.66-1.44	0.896		1.10	0.69-1.74	0.696
BUN, mg/dL >26 vs ≤26	**3.67**	**2.14-6.28**	**<0.001**		4.34	**2.03-9.30**	**<0.001**
Creatinine, mg/dL >1.3/1.1 vs ≤1.3/1.1*	**1.83**	**1.24-2.70**	**0.002**		1.06	0.58-1.93	0.853
Uric Acid, mg/dL >7.5/6.5 vs ≤7.5/6.5*	1.37	0.85-2.20	0.197		1.89	0.99-3.59	0.053
ECOG score							
2	Reference				Reference		
3	1.05	0.68-1.62	0.829		0.80	0.48-1.32	0.374
4	1.40	0.89-2.20	0.144		1.52	0.84-2.72	0.164

BP: Blood Pressure; BMI: Body Mass Index; HDL-C: High-density lipoprotein-Cholesterol; BUN: Blood urine nitrogen; ECOG: Eastern Cooperative Oncology Group.

*Male/Female

**Figure 2:**
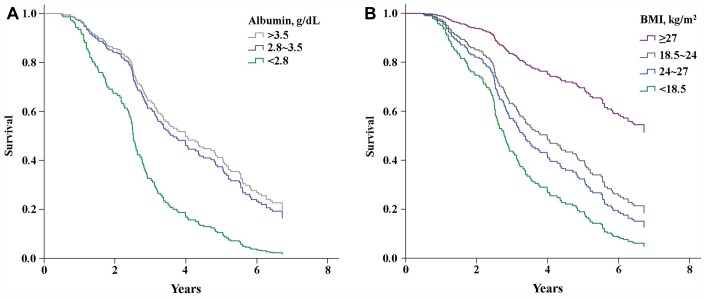
Kaplan-Meier curves showing the effects of serum albumin levels (**A**) (P<0.001) and BMI (**B**) (P<0.001) on survival.

From year 1 of the follow-up period onward, participants with both BMIs <18.5 kg/m^2^ and albumin levels <2.8 mg/dL had a lower survival rate (Figure 2A and 2B). On the other hand, the survival rate did not differ significantly between participants with albumin levels of 2.8–3.5 mg/dL or >3.5 mg/dL. The highest mortality was among participants with albumin levels <2.8 mg/dL and BMIs <18.5 kg/m^2^ (HR = 6.12, 95% CI = 1.85-20.21, *P* = 0.003; Table 3).

**Table 3:** Multivariable adjusted Cox model in elderly patients at different BMIs and serum albumin levels with (A) DBP <90 mmHg or (B) DBP >90 mmHg

**A**												
**BMI, kg/m^2^**												
		**<18.5**			**18.5-24**			**24-27**			**>27**	
	%	**HR**	**95%CI**	**%**	**HR**	**95%CI**	**%**	**HR**	**95%CI**	**%**	**HR**	**95%CI**
Albumin, g/dL												
<2.8	3.8	6.12**	1.85-20.21	4.9	3.40*	1.25-9.28	0.5	2.65	0.29-24.05			
2.8-3.5	14.8	2.55*	0.11-5.87	29.1	1.36	0.66-2.78	11	2.19	0.88-5.47	1.6	1.005	0.18-5.74
>3.5	2.7	4.01*	1.09-14.75	9.9	Reference		4.9	0.81	0.26-2.55	3.3	0.649	0.15-2.75
**B**												
**BMI, kg/m^2^**												
		**<18.5**			**18.5-24**			**24-27**			**>27**	
	**%**	**HR**	**95%CI**	**%**	**HR**	**95%CI**	**%**	**HR**	**95%CI**	**%**	**HR**	**95%CI**
Albumin, g/dL												
<2.8	1.1	12.75**	2.12-76.58	0.5	3.02	1.25-36.19						
2.8-3.5	2.7	1.13	0.27-4.81	3.3	3.45*	1.06-11.19	0.5	4.76	1.47-48.21			
>3.5	0.5	98.39**	4.59-1126.33	1.1	2.58	0.28-23.67	1.6	5.50	1.13-26.80	0.5	5.25	1.49-55.80

*P<0.05, **P<0.01

## DISCUSSION

Low serum albumin levels not only increase the risk of postoperative organ dysfunction, they are also predictive of mortality in the healthy elderly [19, 20]. BMIs are inversely related to mortality in the elderly, and the combination of albumin level and BMI is used to predict risk of mortality in cardiac surgery and dialysis patients [21–23]. Our cohort study demonstrated that among elderly participants with limited performance status (ECOG ≥2), the mortality rate was 6-fold when the serum albumin level was <2.8 mg/dL and BMI was <18.5 kg/m^2^.

### BMI

Low BMI (<18.5 kg/m^2^) is predictive of mortality in the elder population. Large cohort studies of elders in Australia [24], Europe [25], and Taiwan [26] showed that being underweight associates with higher all-cause mortality risk. Similarly, we found an association between lower BMI and increased mortality risk in elders with limited musculoskeletal performance status (Figure 2B). We also found that mortality among participants with BMIs <18.5 kg/m^2^ and serum albumin levels <2.8 g/dL is higher than among those with BMIs <18.5 kg/m^2^ and albumin levels >2.8 g/dL (Table 3A). This is consistent with the finding of Engelman et al. [22], who reported that elders with lower BMIs and serum albumin levels (<2.8 g/dL) showed greater mortality than those with lower BMIs but normal serum albumin levels. This suggests that low BMI alone does not increase the risk of all-cause mortality.

### Albumin

A large cross-sectional longitudinal cohort study of a general elderly population (mean age = 73 years) found an association between albumin levels <3.6 g/dL and higher mortality rates (HR = 2.8, 95% CI = 2.5-3.3) [26]. We found a similar association among limited-performance elders (mean age = 78.8 ± 7.5) when serum albumin levels were <3.5 mg/dL (Figure 2A). In addition, a low serum albumin level is a prognostic factor in cardiovascular disease [27], malnutrition [28], inflammatory reactions [29] and nephrotic syndrome [30] in elders. Similarly, we found that lower serum albumin levels associate with an increased risk of death (HR = 2.54, 95% CI = 1.22-5.30, *P* = 0.013) after adjusting for several potential confounders, including age, renal function, and BP (Table 2). Elderly patients undergoing surgical intervention are at higher risk than younger patients for such postoperative morbidities as pneumonia (OR = 1.76, 95% CI = 1.34-2.33, *P* <0.001) and sepsis (OR = 2.29, 95% CI = 1.22-4.30, *P* = 0.010) [31]. Consequently, a lower serum albumin may reflect physiological impairments such as inflammation, malnutrition or malabsorption in older adults [32]. Importantly, the survival rate among older patients depends not only on the aforementioned physiological impairments, but also on such preexisting conditions as limited performance status, cognitive impairment, and low BMI [33].

### Albumin and BMI

Combined low albumin levels and BMIs in the elderly associate with poor prognoses in several diseases [10, 34–37]. Mechanisms to explain lower serum albumin [35, 38] and BMI [39] may involve chronic inflammation, which appears to be a common factor in various diseases affecting the elderly, including cancer, cardiovascular disease, diabetes, cognitive impairment, frailty, sarcopenia [40, 41]. Compared to individuals without inflammation or obesity, patients exhibiting chronic inflammation have a greater risk of mortality [HR = 2.68, 95%CI = 2.14-3.35] when BMI is <21.5 kg/m^2^ [42]. Moreover, inflammation may be the main cause of reduced serum albumin levels [43], and increasing the BMI can decrease the negative consequences of inflammation [42]. Our finding that mortality was highest when serum albumin was <2.8 g/dL and BMI was <18.5 kg/m^2^ is consistent with those earlier findings (Table 3A).

We also found that the risk of mortality increased about 2-fold in all categories of BMI and albumin when DBP was >90 mmHg (Table 3B). Likewise, Athanase et al. reported that all-cause mortality in the elderly is increased when DBP is >80 mmHg [44]. Thus, hypertension alone can increase the risk of all-cause mortality. However, a recent large prospective study of the general population reported that the combination of low-grade albuminuria and BMI predict mortality more accurately than low-grade albuminuria or BMI alone [11]. Our findings in elders with limited performance differs from those in middle aged individuals reported by Xiong et al. In elders, urinary tract infections and. cardiovascular diseases are common health conditions, and other conditions include musculoskeletal impairment, chronic obstructive pulmonary disease, cognitive impairment, cancer, diabetes mellitus, and inflammatory diseases [45]. Albuminuria can be affected by both physiological and abnormal stressors, such as exercise [46], posture [47], urinary tract infection [48], and myocardial ischemia [49]. Serum albumin is less affected by these conditions. For long-term medical care, therefore, combining BMI, albumin levels, and DBP as health condition indicators may be suitable for elderly individuals with limited performance status

### Strengths and limitations

A key strength of our study is the relatively long follow-up period for elderly individuals with limited performance status. However, there were also several limitations. First, the number of participants was small. Second, the study did not use recent biological data or anthropometric indices. Therefore, changes in those factors could influence the results. Third, the height of some patients could not be measured, so arm length was used to estimate their BMI, which may have introduced bias into our results. Finally, because all of our participants were Taiwanese, our findings may not be applicable to other ethnic groups.

## CONCLUSION

Combined hypoalbuminemia (<2.8 g/dL) and low BMI (<18.5 kg/m^2^) may be a useful prognostic indicator for predicting high risk of mortality in elderly individuals with limited performance status (ECOG score ≥2). These findings could aid in early identification of relevant vulnerable populations.

## MATERIALS AND METHODS

### Participants

The nutritional status of elderly individuals in long-term care facilities (NSELCF) study is an ongoing longitudinal cohort study, which recruited 374 >65-year-old residents in eight long-term care facilities in Taiwan [50]. By the end of the follow-up on December 31, 2009, only 265 participants (men, 43.0%) remained. Of those, 37 were excluded because they had missing anthropometric or laboratory data, and 46 were excluded because they had ECOG scores <2 (Figure 1). Ultimately, 182 individuals were analyzed. Medical ethics approval for participant recruitment and data analyses was obtained from the Institutional Review Board of the China Medical University Hospital. All participants provided written informed consent.

### Measurements

The anthropometric indices included age, sex, weight (to the nearest 0.1 kg), height (to the nearest 0.1 cm), BMI (calculated as weight (kg) / estimated height squared (m^2^)), WC (to the nearest 0.1 cm), blood pressure (in mmHg), and ECOG score. The participants were divided into five categories from 0 (completely active) to 4 (completely disabled). For participants whose height measurement could not be directly measured, height was estimated based on arm span (length from the fingertips of one hand to those of the other hand [51]).

Blood was drawn while patients were in a lying or seated position. In addition to blood counts, the chemistry profile included creatinine, BUN, albumin, fasting glucose, cholesterol, TG, high-density lipoprotein cholesterol (HDL-C), low-density lipoprotein cholesterol (LDL-C), and uric acid levels.

### Mortality

Mortality data were obtained from the Department of Health and Welfare in Taiwan from January 2003 to December 2009.

### Statistical analysis

Multivariable-adjusted Cox proportional hazards models were used to estimate HRs and 95% CIs. Anthropometric and laboratory data were categorized as follows: systolic blood pressure (≤120, 121–139, and ≥140 mmHg); DBP (≤80, 81–89, and ≥90 mmHg), BMI (≤18.5, 24–27, and ≥27 kg/m^2^); hemoglobin (male: <13.7 and ≥13.7 g/dL; female: <11.1 and ≥11.1 g/dL), albumin (>3.5, 2.8–3.5, and <2.8 g/dL), fasting glucose (≤100, 101-125, and ≥126 mg/dL), total cholesterol (>200 and ≤200 mg/dL), TG (>150 and ≤150 mg/dL), HDL-C (male: >40 and ≤40 mg/dL; female: >50 and ≤50 mg/dL), BUN (>26 and ≤26 mg/dL), creatinine (male: >1.3 and ≤1.3 mg/dL; female: >1.1 and ≤1.1 mg/dL), and uric acid (male: >7.5 and ≤7.5 mg/dL; female: >6.5 and ≤6.5 mg/dL); and ECOG score (2, 3, and 4). ANOVA was used for continuous variables and the chi-square test was used for categorical variables at different albumin levels. Effects of albumin and BMI levels on mortality were determined using the Kaplan-Meier method. The mortality risk corresponding to different BMIs and albumin levels was also calculated. All statistical tests were two-sided, and values of *P* <0.05 were considered significant. All statistical analyses were performed using the PC version of the SPSS statistical software (version 21.0; SPSS Inc., Chicago, IL, USA).

## References

[1] Landi F, Onder G, Gambassi G, Pedone C, Carbonin P, Bernabei R. Body mass index and mortality among hospitalized patients. Arch Intern Med. 2000; 160:2641–44. 10.1001/archinte.160.17.2641 10999978

[2] Locher JL, Roth DL, Ritchie CS, Cox K, Sawyer P, Bodner EV, Allman RM. Body mass index, weight loss, and mortality in community-dwelling older adults. J Gerontol A Biol Sci Med Sci. 2007; 62:1389–92. 10.1093/gerona/62.12.1389 18166690 PMC2750037

[3] Song X, Pitkäniemi J, Gao W, Heine RJ, Pyörälä K, Söderberg S, Stehouwer CD, Zethelius B, Tuomilehto J, Laatikainen T, Tabák AG, Qiao Q, Group DS, and DECODE Study Group. Relationship between body mass index and mortality among Europeans. Eur J Clin Nutr. 2012; 66:156–65. 10.1038/ejcn.2011.145 21829217

[4] Sung J, Bochicchio GV, Joshi M, Bochicchio K, Costas A, Tracy K, Scalea TM. Admission serum albumin is predicitve of outcome in critically ill trauma patients. Am Surg. 2004; 70:1099–102. 15663053

[5] Gatta A, Verardo A, Bolognesi M. Hypoalbuminemia. Intern Emerg Med. 2012 (Suppl 3); 7:S193–99. 10.1007/s11739-012-0802-0 23073857

[6] Akirov A, Masri-Iraqi H, Atamna A, Shimon I. Low Albumin Levels Are Associated with Mortality Risk in Hospitalized Patients. Am J Med. 2017; 130:1465.e11–1465.e19. 10.1016/j.amjmed.2017.07.020 28803138

[7] Gotsman I, Shauer A, Zwas DR, Tahiroglu I, Lotan C, Keren A. Low serum albumin: A significant predictor of reduced survival in patients with chronic heart failure. Clin Cardiol. 2019; 42:365–72. 10.1002/clc.23153 30637771 PMC6712335

[8] Feldman MF. Sleisenger and Fordtran’s gastrointestinal and liver disease: Pathophysiology, diagnosis, management. Philadelphia: Saunders Elsevier; 2006.

[9] Levey AS, Coresh J. Chronic kidney disease. Lancet. 2012; 379:165–80. 10.1016/S0140-6736(11)60178-5 21840587

[10] Kimura Y, Yamada M, Kakehi T, Itagaki A, Tanaka N, Muroh Y. Combination of Low Body Mass Index and Low Serum Albumin Level Leads to Poor Functional Recovery in Stroke Patients. J Stroke Cerebrovasc Dis. 2017; 26:448–53. 10.1016/j.jstrokecerebrovasdis.2016.10.008 27856112

[11] Xiong J, Wang J, Zhao J, Zhang L. Association Between Body Mass Index Combined with Albumin: creatinine Ratio and All-cause Mortality in Chinese Population. Sci Rep. 2017; 7:10878. 10.1038/s41598-017-11084-5 28883431 PMC5589898

[12] Kyu HH, Abate D, Abate KH, Abay SM, Abbafati C, Abbasi N, Abbastabar H, Abd-Allah F, Abdela J, Abdelalim A, Abdollahpour I, Abdulkader RS, Abebe M, et al, and GBD 2017 DALYs and HALE Collaborators. Global, regional, and national disability-adjusted life-years (DALYs) for 359 diseases and injuries and healthy life expectancy (HALE) for 195 countries and territories, 1990-2017: a systematic analysis for the Global Burden of Disease Study 2017. Lancet. 2018; 392:1859–922. 10.1016/S0140-6736(18)32335-3 30415748 PMC6252083

[13] Austad SN. The Geroscience Hypothesis: Is It Possible to Change the Rate of Aging? In: Sierra F., Kohanski R. (eds) Advances in Geroscience. Springer, Cham. 2016;1–36. 10.1007/978-3-319-23246-1_1

[14] Oken MM, Creech RH, Tormey DC, Horton J, Davis TE, McFadden ET, Carbone PP. Toxicity and response criteria of the Eastern Cooperative Oncology Group. Am J Clin Oncol. 1982; 5:649–55. 10.1097/00000421-198212000-00014 7165009

[15] Yourman LC, Lee SJ, Schonberg MA, Widera EW, Smith AK. Prognostic indices for older adults: a systematic review. JAMA. 2012; 307:182–92. 10.1001/jama.2011.1966 22235089 PMC3792853

[16] Thomas JM, Cooney LM Jr, Fried TR. Systematic review: health-related characteristics of elderly hospitalized adults and nursing home residents associated with short-term mortality. J Am Geriatr Soc. 2013; 61:902–11. 10.1111/jgs.12273 23692412 PMC4059538

[17] Tabue-Teguo M, Kelaiditi E, Demougeot L, Dartigues JF, Vellas B, Cesari M. Frailty Index and Mortality in Nursing Home Residents in France: Results From the INCUR Study. J Am Med Dir Assoc. 2015; 16:603–06. 10.1016/j.jamda.2015.02.002 25769962

[18] Flacker JM, Kiely DK. Mortality-related factors and 1-year survival in nursing home residents. J Am Geriatr Soc. 2003; 51:213–21. 10.1046/j.1532-5415.2003.51060.x 12558718

[19] Rady MY, Ryan T, Starr NJ. Clinical characteristics of preoperative hypoalbuminemia predict outcome of cardiovascular surgery. JPEN J Parenter Enteral Nutr. 1997; 21:81–90. 10.1177/014860719702100281 9084010

[20] Klonoff-Cohen H, Barrett-Connor EL, Edelstein SL. Albumin levels as a predictor of mortality in the healthy elderly. J Clin Epidemiol. 1992; 45:207–12. 10.1016/0895-4356(92)90080-7 1569417

[21] Vashistha T, Mehrotra R, Park J, Streja E, Dukkipati R, Nissenson AR, Ma JZ, Kovesdy CP, Kalantar-Zadeh K. Effect of age and dialysis vintage on obesity paradox in long-term hemodialysis patients. Am J Kidney Dis. 2014; 63:612–22. 10.1053/j.ajkd.2013.07.021 24120224 PMC3969454

[22] Engelman DT, Adams DH, Byrne JG, Aranki SF, Collins JJ Jr, Couper GS, Allred EN, Cohn LH, Rizzo RJ. Impact of body mass index and albumin on morbidity and mortality after cardiac surgery. J Thorac Cardiovasc Surg. 1999; 118:866–73. 10.1016/S0022-5223(99)70056-5 10534692

[23] Feingold E, Adams J, Penprase B, Tubie B. Effect of body mass index and albumin on mortality rates for adult African-American hemodialysis patients. J Am Assoc Nurse Pract. 2015; 27:637–45. 10.1002/2327-6924.12236 25761048

[24] Flicker L, McCaul KA, Hankey GJ, Jamrozik K, Brown WJ, Byles JE, Almeida OP. Body mass index and survival in men and women aged 70 to 75. J Am Geriatr Soc. 2010; 58:234–41. 10.1111/j.1532-5415.2009.02677.x 20370857

[25] de Hollander EL, Van Zutphen M, Bogers RP, Bemelmans WJ, De Groot LC. The impact of body mass index in old age on cause-specific mortality. J Nutr Health Aging. 2012; 16:100–06. 10.1007/s12603-011-0077-622238008

[26] Wu CY, Hu HY, Huang N, Chou YC, Li CP, Chou YJ. Albumin levels and cause-specific mortality in community-dwelling older adults. Prev Med. 2018; 112:145–51. 10.1016/j.ypmed.2018.04.015 29649489

[27] Weijenberg MP, Feskens EJ, Souverijn JH, Kromhout D. Serum albumin, coronary heart disease risk, and mortality in an elderly cohort. Epidemiology. 1997; 8:87–92. 9116102 10.1097/00001648-199701000-00014

[28] Hannan JL, Radwany SM, Albanese T. In-hospital mortality in patients older than 60 years with very low albumin levels. J Pain Symptom Manage. 2012; 43:631–37. 10.1016/j.jpainsymman.2011.04.009 21925833

[29] Hedlund JU, Hansson LO, Ortqvist AB. Hypoalbuminemia in hospitalized patients with community-acquired pneumonia. Arch Intern Med. 1995; 155:1438–42. 10.1001/archinte.1995.00430130132014 7794094

[30] Harris RC, Ismail N. Extrarenal complications of the nephrotic syndrome. Am J Kidney Dis. 1994; 23:477–97. 10.1016/S0272-6386(12)80369-6 8154483

[31] Morotti A, Marini S, Lena UK, Crawford K, Schwab K, Kourkoulis C, Ayres AM, Edip Gurol M, Viswanathan A, Greenberg SM, Anderson CD, Rosand J, Goldstein JN. Significance of admission hypoalbuminemia in acute intracerebral hemorrhage. J Neurol. 2017; 264:905–11. 10.1007/s00415-017-8451-x 28283821 PMC7436338

[32] Walston J, McBurnie MA, Newman A, Tracy RP, Kop WJ, Hirsch CH, Gottdiener J, Fried LP, and Cardiovascular Health Study. Frailty and activation of the inflammation and coagulation systems with and without clinical comorbidities: results from the Cardiovascular Health Study. Arch Intern Med. 2002; 162:2333–41. 10.1001/archinte.162.20.2333 12418947

[33] Bo M, Massaia M, Raspo S, Bosco F, Cena P, Molaschi M, Fabris F. Predictive factors of in-hospital mortality in older patients admitted to a medical intensive care unit. J Am Geriatr Soc. 2003; 51:529–33. 10.1046/j.1532-5415.2003.51163.x 12657074

[34] Phung DT, Wang Z, Rutherford S, Huang C, Chu C. Body mass index and risk of pneumonia: a systematic review and meta-analysis. Obes Rev. 2013; 14:839–57. 10.1111/obr.12055 23800284

[35] Viasus D, Garcia-Vidal C, Simonetti A, Manresa F, Dorca J, Gudiol F, Carratalà J. Prognostic value of serum albumin levels in hospitalized adults with community-acquired pneumonia. J Infect. 2013; 66:415–23. 10.1016/j.jinf.2012.12.007 23286966

[36] Mafra D, Farage NE, Azevedo DL, Viana GG, Mattos JP, Velarde LG, Fouque D. Impact of serum albumin and body-mass index on survival in hemodialysis patients. Int Urol Nephrol. 2007; 39:619–24. 10.1007/s11255-007-9201-2 17450420

[37] Courtney D, Moloney B, Lowery A, Kerin M. Patients Nutritional Status: BMI and serum albumin as a predictive indicator of survival in patients with metastatic breast cancer. Eur J Surg Oncol. 2017; 43:S29–30. 10.1016/j.ejso.2017.01.120

[38] Hong X, Yan J, Xu L, Shen S, Zeng X, Chen L. Relationship between nutritional status and frailty in hospitalized older patients. Clin Interv Aging. 2019; 14:105–11. 10.2147/CIA.S189040 30666096 PMC6330965

[39] Veronese N, Cereda E, Solmi M, Fowler SA, Manzato E, Maggi S, Manu P, Abe E, Hayashi K, Allard JP, Arendt BM, Beck A, Chan M, et al. Inverse relationship between body mass index and mortality in older nursing home residents: a meta-analysis of 19,538 elderly subjects. Obes Rev. 2015; 16:1001–15. 10.1111/obr.12309 26252230

[40] Sanada F, Taniyama Y, Muratsu J, Otsu R, Shimizu H, Rakugi H, Morishita R. Source of Chronic Inflammation in Aging. Front Cardiovasc Med. 2018; 5:12. 10.3389/fcvm.2018.00012 29564335 PMC5850851

[41] Accardi G, Caruso C. Immune-inflammatory responses in the elderly: an update. Immun Ageing. 2018; 15:11. 10.1186/s12979-018-0117-8 29507595 PMC5833087

[42] Stenvinkel P, Gillespie IA, Tunks J, Addison J, Kronenberg F, Drueke TB, Marcelli D, Schernthaner G, Eckardt KU, Floege J, Froissart M, Anker SD, Committee AR, and ARO Steering Committee. Inflammation Modifies the Paradoxical Association between Body Mass Index and Mortality in Hemodialysis Patients. J Am Soc Nephrol. 2016; 27:1479–86. 10.1681/ASN.2015030252 26567245 PMC4849822

[43] Kaysen GA, Dubin JA, Müller HG, Rosales L, Levin NW, Mitch WE, and HEMO Study Group NIDDK. Inflammation and reduced albumin synthesis associated with stable decline in serum albumin in hemodialysis patients. Kidney Int. 2004; 65:1408–15. 10.1111/j.1523-1755.2004.00520.x 15086482

[44] Protogerou AD, Safar ME, Iaria P, Safar H, Le Dudal K, Filipovsky J, Henry O, Ducimetière P, Blacher J. Diastolic blood pressure and mortality in the elderly with cardiovascular disease. Hypertension. 2007; 50:172–80. 10.1161/HYPERTENSIONAHA.107.089797 17515449

[45] Odding E, Valkenburg HA, Stam HJ, Hofman A. Determinants of locomotor disability in people aged 55 years and over: the Rotterdam Study. Eur J Epidemiol. 2001; 17:1033–41. 10.1023/A:1020006909285 12380718

[46] Poortmans JR. Postexercise proteinuria in humans. Facts and mechanisms. JAMA. 1985; 253:236–40. 10.1001/jama.1985.03350260088032 3965775

[47] Montagna G, Buzio C, Calderini C, Quaretti P, Migone L. Relationship of proteinuria and albuminuria to posture and to urine collection period. Nephron. 1983; 35:143–44. 10.1159/000183064 6621758

[48] Carter JL, Tomson CR, Stevens PE, Lamb EJ. Does urinary tract infection cause proteinuria or microalbuminuria? A systematic review. Nephrol Dial Transplant. 2006; 21:3031–37. 10.1093/ndt/gfl373 16861738

[49] Berton G, Cordiano R, Palmieri R, Cucchini F, De Toni R, Palatini P. Microalbuminuria during acute myocardial infarction; a strong predictor for 1-year mortality. Eur Heart J. 2001; 22:1466–75. 10.1053/euhj.2000.2582 11482920

[50] Lin WY, Huang HY, Liu CS, Li CI, Lee SD, Lin CC, Huang KC. A hospital-based multidisciplinary approach improves nutritional status of the elderly living in long-term care facilities in middle Taiwan. Arch Gerontol Geriatr. 2010 (Suppl 1); 50:S22–26. 10.1016/S0167-4943(10)70007-8 20171451

[51] Weinbrenner T, Vioque J, Barber X, Asensio L. Estimation of height and body mass index from demi-span in elderly individuals. Gerontology. 2006; 52:275–81. 10.1159/000094608 16974098

